# Diaphyseal humeral fractures and intramedullary nailing: Can we improve outcomes?

**DOI:** 10.4103/0019-5413.67117

**Published:** 2011

**Authors:** Christos Garnavos

**Affiliations:** Department of Orthopedics, Evangelismos General Hospital, Athens, Greece

**Keywords:** Intramedullary nailing, diaphyseal humeral fracture, humerus, fixed nail, bio-nail

## Abstract

While intramedullary nailing has been established as the treatment of choice for diaphyseal fractures of the femur and tibia, its role in the management of diaphyseal humeral fractures remains controversial. The reasons include not only the complicated anatomy and unique biomechanical characteristics of the arm but also the fact that surgical technique and nail designs devised for the treatment of femoral and tibial fractures are being transposed to the humerus. As a result there is no consensus on many aspects of the humeral nailing procedure, e.g., the basic nail design, nail selection criteria, timing of the procedure, and the fundamental principles of the surgical technique (e.g., antegrade/retrograde, reamed/unreamed, and static/dynamic). These issues will be analyzed and discussed in the present article. Proposals aiming to improve outcomes include the categorization of humeral nails in two distinct groups: “fixed” and “bio”, avoidance of reaming for the antegrade technique and utilization of “semi-reaming” for the retrograde technique, guidelines for reducing complications, setting the best “timing” for nailing and criteria for selecting the most appropriate surgical technique (antegrade or retrograde). Finally, suggestions are made on proper planning and conducting clinical and biomechanical studies regarding the use of intramedullary nailing in the management of humeral shaft fractures.

## INTRODUCTION

*Intramedullary splintage, either with a nail or with Rush rods, has virtually no place in the treatment of acute humeral fractures. Not only can the nail or Rush rod or Küntscher nail easily lead to damage and stiffness of the shoulder joint because of interference with the rotator cuff, but they also fail to provide sufficient stability’*.^1^ This was an important statement made two decades ago. Although in the last twenty years we witnessed enormous changes and significant improvements in the philosophy and treatment methods of fractures in general, the treatment of choice for acute, uncomplicated diaphyseal humeral fractures remains non-operative.^2^–^8^ Furthermore, when surgical treatment is contemplated, it is still generally believed that intramedullary nailing may not be the best choice.^3^,^9^–^18^

It is paradox why a surgical technique, so successful in the treatment of diaphyseal femoral and tibial fractures cannot produce similar results when applied in the humerus. A possible explanation is that the complex anatomy and the unique biomechanical characteristics of the humerus are overlooked. Furthermore, there has been no consensus so far regarding either the fundamental principles of the surgical technique (e.g., antegrade/retrograde, reamed/unreamed, static/dynamic) or important technical aspects (such as the basic nail design and nail selection criteria). The present manuscript deals with these problems and other controversial issues and, furthermore, tries to define the guidelines and principles that should be considered whenever intramedullary nailing is selected for the treatment of diaphyseal humeral fractures.

## TECHNICAL PROBLEMS

### Antegrade nailing

#### a. Violation of the rotator cuff

Violation of the rotator cuff during antegrade humeral nailing has been considered to be responsible for suboptimal clinical outcomes and discomfort in the shoulder joint.^6^,^15^,^19^–^21^ Retrograde nailing was mainly introduced as an alternative technique to bypass this problem.^22^–^24^ Recent reports have proposed modifications of the antegrade surgical technique or introduced ‘sophisticated’ nail designs in order to overcome this problem but so far these proposals have not been validated with further studies.^25^–^27^

Interestingly, shoulder joint problems have been reported in patients who, following humeral shaft fractures, were treated by therapeutic modules that did not interfere with the shoulder anatomy, e.g., bracing or plating. In these cases, prolonged immobilization (either before plating or during bracing) was considered to be the precipitating factor.^6^,^28^–^31^ There has also been a report about patients with diaphyseal humeral fractures who were treated with retrograde nailing without delay but developed stiffness and discomfort of the shoulder joint.^32^ Although their symptoms subsided several weeks later with intensive physiotherapy, the authors could not explain how was the problem created. These reports of shoulder impairment occurring even when there has been no direct surgical intervention to the joint suggest that shoulder pathology may occur after a humeral shaft fracture regardless of the treatment method, and antegrade intramedullary nailing may not always be the precipitating cause of shoulder discomfort and functional impairment.

A recent study^32^ reported 45 patients who sustained isolated traumatic humeral shaft fractures and were treated with antegrade intramedullary nailing. All regained full painless range of shoulder joint motion. The authors proposed that a simple in-out incision on the rotator cuff, use of unreamed technique, and meticulous repair at the end of the procedure contributed to the uneventful shoulder joint functional recovery in these cases.

#### b. Soft tissue injury around the shoulder

**Figure 1:**
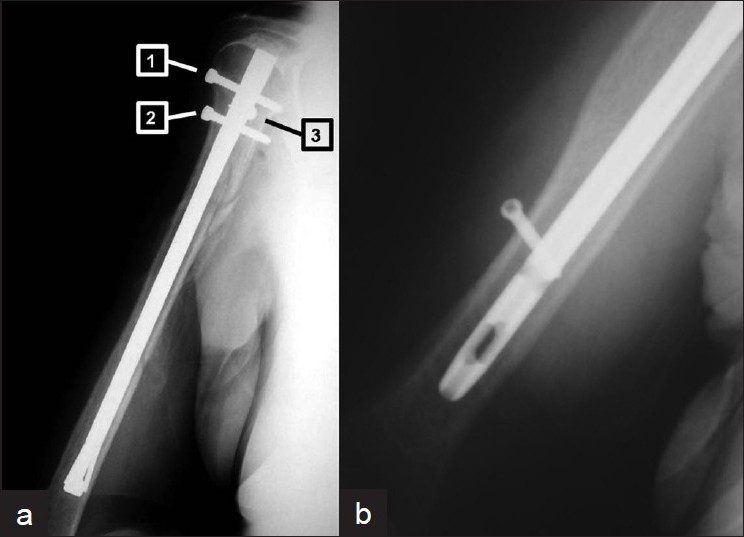
(a) X-ray of the arm including the shoulder joint (anteroposterior view) depicting that proximal locking screws could injure vulnerable soft tissues around the shoulder during antegrade nailing: Screw [1] could damage the circumflex humeral artery, screw [2] could damage the axillary nerve and screw [3] could damage the long head of biceps. (b) X-ray of the lower arm (lateral view) depicting a “miss a hole” situation after the use of a targeting device

Vulnerable structures around the shoulder that could be injured during antegrade intramedullary nailing include the axillary nerve, the circumflex artery, the long head of biceps, and the deltoid. These structures are usually injured by the proximal locking bolts, and modern targeting devices have not abolished this complication^33^–^40^ [Figure 1a].

The incidence of injuries to the long head of the biceps and the axillary nerve could be reduced with the avoidance of the anteroposterior locking screw that many nails provide. Retrograde nails that use screws for the proximal interlock reduce but do not abolish the incidence of injury to vulnerable soft tissues around the shoulder girdle.^37^ Additionally, the use of antegrade or retrograde nails that do not use locking bolts for the proximal interlock (e.g., True-Flex nail, Marchetti nail, Fixion nail, or Garnavos nail) do not cause these complications, though these nails could increase the risk of problems with the fracture union process due to reduced stability at the fracture site.

#### c. Distal interlocking

Unlike in the femur and tibia, distal interlocking of an antegrade humeral nail is considered difficult and time consuming as a lateral view of the humerus cannot be easily obtained with the image intensifier. Furthermore, the narrow locking holes of humeral nails and the ‘slippery’ bony surface at the distal humerus make distal interlocking even more challenging.^41^

The technically ‘easy’ to insert anteroposterior distal locking screw requires a careful open approach to the anterior supracondylar area of the distal humerus due to the presence of important neurovascular structures. Insertion of a locking screw from lateral to medial, apart from being technically more difficult, bears the danger of injury to the radial and/or the lateral cutaneous nerves. Such a lateromedial locking screw could be inserted more easily with the arm resting in abduction and internal rotation on a radiolucent support to facilitate viewing with the image intensifier (though there is the danger of losing reduction of the fracture). An open approach for this lateromedial screw insertion has also been recommended.^42^,^43^

One non-cannulated screw is usually enough for distal locking of an antegrade humeral nail and its insertion could be facilitated with the opening of a starting hole at the near cortex, with a Steinman pin to engage the tip of the drill bit.

Targeting devices, which some manufacturers provide, have been tried for the distal interlock of antegrade nails. However, their success has not been consistent, possibly due to their long length and the narrow nail diameter^42^,^44^,^45^ [Figure 1b].

#### d. Soft tissue injury around the elbow

Lateromedial screw insertion for the distal interlock of antegrade nails is associated with a significant risk of injury to the radial nerve or the lateral cutaneous nerve in the supracondylar area.^33^,^35^,^42^,^43^,^46^–^48^ Most authors recommend an open approach under ‘direct vision’ to the supracondylar area, with exploration and confirmation of the avoidance of injury to the radial and lateral cutaneous nerves. Inevitably, operating time is prolonged and the minimal invasiveness of the intramedullary nailing procedure is jeopardized. One of the reasons for the introduction of retrograde humeral nailing is to avoid this problem, as most retrograde nails are locked with postero-anterior screws that are inserted in a ‘safe’ area. Alternatively, new antegrade humeral nails that do not use screws for the distal interlock – and thus avoid iatrogenic injury to the radial and lateral cutaneous nerves (e.g., True-Flex, Marchetti, or Garnavos) – can be used, though this would be at the expense of optimal fracture stability.

Problems with the median nerve and brachial artery have not been reported in the literature, either because surgeons avoid antero-posterior distal locking of antegrade nails or because they use the ‘open locking’ technique.

### Retrograde nailing

#### a. Eccentric nail insertion

**Figure 2:**
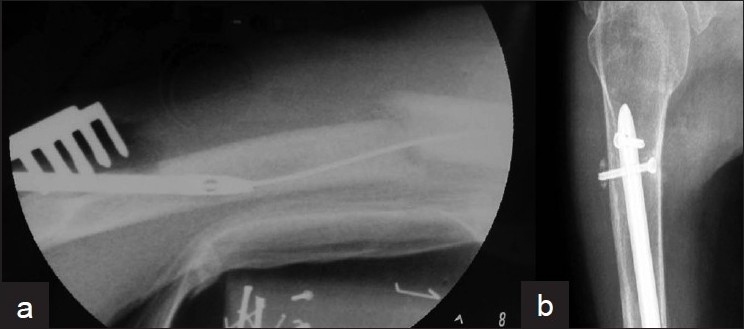
(a) Lateral radiograph of lower arm depicting that the danger of a supracondylar fracture during retrograde nailing is substantial, due to entry hole made for nail insertion. (b) X-ray of the upper arm (lateral view) depicting that locking proximally a retrograde nail could be facilitated if performed below the surgical neck of the humeral head

During retrograde nailing the olecranon does not allow nail insertion in line with the humeral canal. The nail is therefore directed diagonally from the posteriorly located entrance hole towards the anterior cortex, and this eccentric insertion increases the danger of a supracondylar fracture around the entry portal”^10^,^36^,^49^ [Figure 2a]. The occurrence of this devastating complication can be avoided by making a broad entry portal, oval in shape, with dimensions of at least 1 cm × 2 cm in the axis of humerus. Careful hand reaming of the distal humeral canal could facilitate nail insertion. Finally, the use of nonrigid nails (e.g., titanium) should further reduce the incidence of iatrogenic fractures in the supracondylar area.^10^,^44^

#### b. Proximal interlock

While distal interlock of retrograde nails is usually straightforward, either under direct vision or with accurate/short targeting devices in a postero-anterior ‘safe’ direction, the proximal interlock is associated with significant difficulties, similar to that seen with distal interlock in antegrade nailing. These difficulties relate to the problematic visualization of the proximal humerus with the image intensifier and the demanding ‘free-hand’ technique.^32^,^44^,^50^ It is recommended that the proximal interlock of retrograde nails should be performed below the area of the surgical neck of the humeral head [Figure 2b]. In this way, the vulnerable soft tissues around the shoulder are avoided and visualization with the image intensifier becomes easier. However, this can be achieved only if the fracture does not extend close to the humeral head.

As targeting devices cannot provide reliable assistance, the ‘free-hand’ technique remains the ’gold standard’ for the proximal interlock of retrograde nails, but it requires significant experience. With regard to the provision of adequate stability to the fracture site, the results with retrograde nails that provide proximal locking facility without the use of locking bolts (e.g., Marchetti nail, Fixion nail, or Halder nail) have been equivocal.^44^,^51^–^54^

#### c. Soft tissue injury around the shoulder

Apart from being difficult, proximal interlock with screws in retrograde nailing could damage the vulnerable soft tissues around the shoulder girdle, for example, the axillary nerve. Although this danger could be considered theoretical, there has been a recent report of its occurrence.^37^

## DEBATABLE ISSUES

### Which nail?

**Figure 3:**
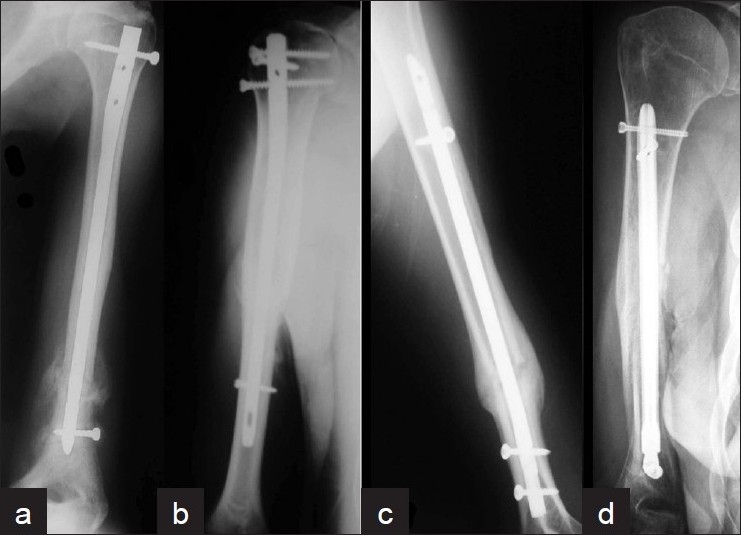
Plain radiographs of arm of “Fixed” nails (anteroposterior view) showing (a) Uniflex (Biomet), (b) Polarus (Acumed), (c) UHN (Synthes), (d) Garnavos (MERETE)

**Figure 4:**
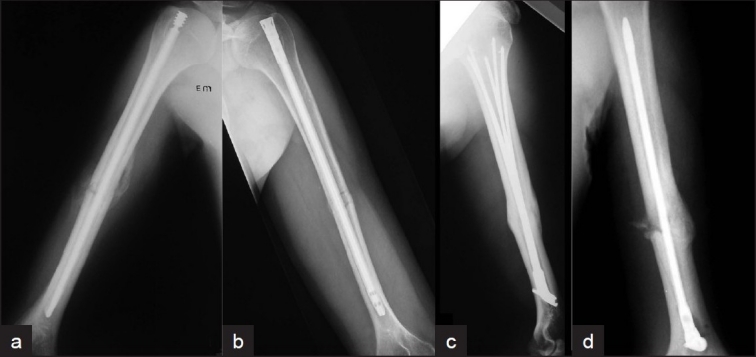
Plain radiographs of arm (anteroposterior a, b and lateral c, d views) of “Bio” nails showing (a) True-Flex (Encore), (b) Garnavos antegrade (MERETE), (c) Marchetti (Zimmer), (d) Garnavos retrograde (MERETE)

Intramedullary nailing of humerus started with Rush pins, Ender nails, and Küntscher nails of small sizes.^15^,^55^,^56^ Over the recent years various nail designs appeared. Some were smaller copies of nails already used for the management of femoral and tibial fractures while others were specially designed to accommodate the anatomical and biomechanical peculiarities of the humerus. This diversity in identities and capabilities of different nail designs has been somehow confusing, as the advantages and disadvantages of each nail have not been clearly defined. As a solution to the problem, it has been proposed that humeral nails should be categorized as ‘fixed nails‘ and ’bio nails’, depending on the interlocking mechanism of their end opposite to the entry portal. According to this proposal, humeral nails that use screws for locking their distal end (distal interlock in antegrade nailing or proximal interlock in retrograde nailing) constitute the ‘fixed’ group (e.g., UHN Synthes, Polarus Acumed, Uniflex Biomet, T2 Stryker) [Figure 3] while nails that provide this interlock with means other than screws constitute the ‘bio’ group (e.g., Marchetti-Vincenzi Zimmer, True-Flex Encore, Fixion Disc-O-Tec, and Garnavos MERETE) [Figure 4]. This categorization could help in clearing up certain points of confusion about humeral nailing, set useful guidelines regarding nail selection criteria, help in deciding the appropriate surgical technique for each case and verify the type of the most suitable rehabilitation protocol. In details, as locking bolts provide adequate stability regardless of fracture location, ‘fixed’ nails can be inserted with either antegrade or retrograde technique without significant consequences to fracture stability and healing process; the selection of one or the other surgical technique depends only on nail design and surgeon preference. On the contrary, fracture location plays an important role in selecting the antegrade or retrograde technique for the insertion of a ‘bio’-nail, as these nails offer alternative distal locking mechanism. Therefore, ‘bio’ nails that stabilise the other end of the humerus by expansion, divergent rods, special design etc, should be inserted either via a proximal (antegrade) or a distal (retrograde) portal, that would allow this mechanism to act as much as possible. This will happen if the part of the nail (that act as ‘distal locking mechanism’) lies within the longest intact humeral segment. In addition, it has been shown that a fracture is stabilised better if the most secure locking (which in ‘bio’ nails is the locking at the entry portal) is closer to the fracture site.^57^,^58^ For those two reasons a ‘bio’ nail should be inserted with the antegrade technique if the fracture is located in the proximal humeral diaphysis while the retrograde technique should be preferred for distal humeral shaft fractures. It could be argued that the terms ‘static’ and ‘dynamic’ could be used instead of ‘fixed’ and ‘bio’. However, ‘static’ or ‘dynamic’ refer to nailing techniques and the options of locking or non-locking a nail. Terms ‘fixed’ or ‘bio’ characterize the design of a nail and indicate if the locking facility is offered with or without screws. ‘Fixed’ nails could withstand a more intensive physiotherapy program, while with ‘bio’-nailing iatrogenic neurovascular complications are reduced at the expense of fracture stability. Finally, the duration of the operative procedure should be less with ‘bio’-nails as screws are not used for distal or proximal (depending on the operative technique) interlocking.^44^

### Antegrade or retrograde?

Since the antegrade procedure has been considered responsible for postoperative shoulder problems due to the intra-articular entry portal and the violation of the rotator cuff, it could be argued that this method should be abandoned and that all humeral nailing procedures would better be undertaken with the retrograde technique. However, there are cases where antegrade humeral nailing is irreplaceable, such as in the polytrauma patient who cannot be positioned prone to undergo retrograde nailing. In addition, the antegrade technique offers easier access to the humeral canal and easier handling of the image intensifier. The technique, being less time consuming, is also preferred by anaesthesiologists.

According to a study by Cheng and Lin (2008), antegrade and retrograde ‘fixed’ nailing have similar treatment results, including healing rate and eventual functional recovery for middle third humeral fractures. However, it is recommended that retrograde (‘fixed’) nailing should be used in patients with wide medullary canal or pre-existing shoulder problems and antegrade (‘fixed’) nailing in patients of young age or those with a small medullary canal.^59^

**Figure 5:**
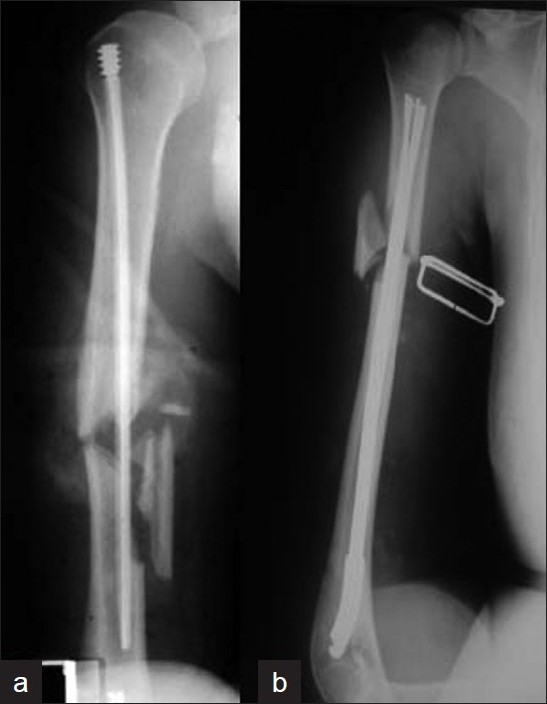
(a) X-ray of arm (anteroposterior view) depicting an example of inappropriate antegrade “Bio” nailing. This humeral nail (True-Flex, Encore) should be used in more proximal humeral fractures, as its locking facility depends on the nail-endosteum contact within the distal fragment. (b) Plain radiograph of arm (anteroposterior view) depicting inappropriate retrograde “Bio” nailing. This humeral nail (Marchetti- Vincenzi, Zimmer) should had been chosen longer in order to allow expansion of the rods within the humeral head

There is no such study on ‘bio’-nails. However, as has been already stated, for biomechanical reasons (better fracture stability) the entry portal of a ‘bio’-nail should be near to the fracture site.^57^,^58^ Therefore, with ‘bio’-nailing, fractures of the proximal half of the humerus should be treated with the antegrade technique, while fractures of the distal half of the humerus should be treated with the retrograde technique. This principle means that ‘bio’-nails that cannot be inserted with both techniques should not be suitable for all fracture patterns (for example the Marchetti-Vicenzi nail, that can be inserted only with the retrograde technique, should not be used in proximal diaphyseal humeral fractures) [Figure 5]. This statement could explain the limited success of ‘bio’-nailing so far, as the significant advantage that these nails offer (of a locking facility without screws) seems to overshadow their limitations, which are related to their inferior biomechanical properties.

### To ream or not to ream?

Reaming plays an important role in the management of femoral and tibial fractures with intramedullary nailing, as the enlarged canal allows the insertion of a wider nail that can offer stronger fixation and facilitate earlier weight-bearing. Humerus, being a non-weight-bearing bone, does not need the widest/strongest nail. In addition, during antegrade humeral nailing, reaming could cause further injury to the rotator cuff with the repeated insertion and withdrawal of several sharp reamers. Furthermore, the rotator cuff could act as a filter for the by-products of reaming and their accumulation underneath may play a role in the pathogenesis of problems that some patients experience postoperatively.^41^,^44^,^60^ Heat-induced segmental necrosis and surgical emphysema have been reported as result of the reaming process during antegrade humeral nailing.^61^–^63^ Finally, although this is only theoretical, there is always a possibility of radial nerve injury from the reamers if a comminuted fracture has occurred at the area where the nerve is in close proximity with the humeral diaphysis.

During retrograde nailing, reaming up to the fracture site may facilitate the eccentric nail insertion without significant consequences as there are not any important vulnerable structures that could be injured by the reamers in the area. Therefore, it could be proposed that reaming should be avoided during antegrade nailing, while careful reaming of the distal humeral segment (semi-reaming) could be recommended during retrograde nailing.

### Timing

Intramedullary nailing of femoral and tibial fractures is usually performed as soon as possible after the injury. Bone healing is promoted by the osteoinductive properties of reaming by-products. In addition, if there are problems with the union process, the surgeon has the option to dynamize the fracture by either converting the nailing from static to dynamic and/or compressing the fracture with the patient’s body weight. In contrast, intramedullary nailing of humeral fractures is not usually performed soon after the injury, as nailing frequently follows failed attempts of conservative treatment. As a result, nailing cannot take advantage of fresh fracture hematoma. In addition, as mentioned earlier, reaming that could help the union process is not recommended in the humerus. Finally, the fracture cannot be effectively dynamized as humerus is not a weight bearing bone. For all these reasons, intramedullary nailing of humeral shaft fractures should be performed sooner than later in order to take advantage of the fresh fracture haematoma and minimize the possibility of healing problems and poor outcomes.

## CLINICAL AND BIOMECHANICAL STUDIES

Over the years, humeral nailing has been considered as a homogenous procedure, regardless of the type of nail or the surgical technique used.^64^,^65^ However, if we bear in mind the vast differences between ‘fixed’ and ‘bio’-nails or antegrade and retrograde techniques, it becomes obvious that published data about humeral nailing, in general, may be misleading. It may not be scientifically sound to compare ‘nailing’ to other treatment modalities without clarifying if the nail is ‘fixed’ or ‘bio’ and the technique antegrade or retrograde. Furthermore, it may be similarly misleading to compare ‘fixed’ nails and ‘bio’-nails in biomechanical studies^66^,^67^ as, by definition, ‘fixed’ nails offer superior stability compared to ‘bio’-nails both *in vivo* and *in vitro*. Therefore, comparative biomechanical studies will be in favor of ‘fixed’ nails. The advantages offered by ‘bio’-nails *in vivo* cannot be reproduced and tested *in vitro*. Consequently, it could be proposed that biomechanical studies should compare nails with similar biomechanical properties. At the bottom line what really matters is the efficacy of an implant to facilitate uneventful fracture union, with low morbidity and rapid recovery.

## CONCLUSIONS

Conservative management is still the treatment of choice for diaphyseal humeral fractures, as operative techniques have not yet produced a persuasive proposal of a treatment method that offers the benefits of minimal invasiveness along with high union and low complication rates, and thus allow rapid shoulder and elbow joint functional recovery and prompt return to work and activities. Intramedullary nailing offers these advantages in the treatment of diaphyseal fractures of the femur and tibia but it has not yet produced similar results in the upper limb. It seems that more time is needed to reach a consensus about the important issues that have been discussed. A deep understanding of the anatomical and biomechanical characteristics of the humerus is essential for arriving at decisions about important issues such as nail selection criteria, operative technique, and a rehabilitation program, whenever intramedullary nailing of diaphyseal humeral fractures is contemplated. A useful guideline that could improve the results of intramedullary nailing in the management of diaphyseal humeral fractures is that a ‘fixed’ nail can be inserted with both antegrade and retrograde techniques regardless of the fracture pattern and location. On the contrary, fracture location could play an important role in the usefulness of ‘bio’ nails as these nails are more effective if their entry portal (antegrade or retrograde technique) is closer to the fracture site. Therefore, antegrade ‘bio’-nailing should be performed for fractures occurring in the proximal half of the humeral diaphysis, while retrograde ‘bio’-nailing should be preserved for fractures located in the distal half of the humeral diaphysis.
